# Radiation- and age-related vascular dysfunction as an early indicator of cardiovascular risk: a long-term study in the ApoE^−/−^ mouse model of atherosclerosis

**DOI:** 10.1186/s40959-025-00395-6

**Published:** 2025-10-21

**Authors:** Bettina Habelt, Maximiliano Cuevas, Wolfgang Dörr

**Affiliations:** 1 Dept. Radiation Oncology, Medical Faculty Carl Gustav Carus, Dresden University of Technology, Fetscherstr. 74, Dresden, 01307 Germany; 2 Dept. Clinical Sensoring and Monitoring, Medical Faculty Carl Gustav Carus, Dresden University of Technology, Fetscherstr. 74, Dresden, 01307 Germany; 3 Dept. Radiation Oncology and CD Laboratory for Medical Radiation Research for Radiation Oncology, Medical University/AKH Vienna, Waehringer Guertel 18-20, Vienna, A-1090 Austria; 4 Department of Psychiatry & Psychotherapy, Medical Faculty Carl Gustav Carus, Dresden University of Technology, Dresden, Germany

**Keywords:** Radiation, Cardiovascular disease, Arterial stiffness, Endothelial dysfunction, Optical coherence tomography

## Abstract

**Background:**

Despite advances in radiotherapeutic techniques, radiation-induced cardiovascular diseases (CVD) remain a leading but often underrecognized cause of morbidity and mortality in cancer survivors. Radiation exposure can trigger a broad spectrum of cardiotoxic effects yet clinical awareness and strategies for managing these long-term complications remain limited. Among emerging indicators of vascular dysfunction, measures of vascular flexibility offer key biomarkers for assessing vascular compliance and cardiovascular risk.

**Methods:**

The present study hence investigated age- and dose-dependent effects of local irradiation on vascular function of the murine Arteria saphena in C57BL/6 wild-type and atherosclerosis-prone apolipoprotein E-knockout (ApoE^−/−^) mice, a well established model for human CVD. Pathological effects of irradiation on vascular function of the A. saphena were assessed using in vivo Optical Coherence Tomography. Vascular flexibility in terms of arterial diameters and speed of diameter changes during vasoconstriction and vasodilation were recorded one day and 3, 6, 9, 12, or 18 months following irradiation with single doses of 2, 5, 8, 10, 16 Gy.

**Results:**

Baseline arterial diameters declined with age in both strains, with earlier onset in ApoE^−/−^ mice. Significant interactions with radiation dose indicate greater radiation sensitivity in ApoE^−/−^ mice and additive effects of radiation and aging in both strains. Vasoconstriction halved arterial diameters in wild-type and more so in ApoE^−/−^ mice, reflecting an enhanced vasoconstrictive response that diminished after 16 Gy. Contractility was found to be age-dependent, peaking between 6 and 12 months post-irradiation, while time to half-maximal constriction remained unchanged across conditions. Maximal vasodilation ranged from 1.2 to 2 × baseline, initially higher in ApoE^−/−^ mice but declining earlier with age than in wildtype mice. ApoE^−/−^ mice exhibited more sustained vasodilation, which progressively slowed with age and higher radiation doses in both strains.

**Conclusion:**

Both mouse strains exhibited marked age-related vascular changes, with ApoE^−/−^ mice showing greater radiation sensitivity. The combined effects of aging and radiation were most prominent in reduced arterial diameters at baseline and after vasoconstriction, along with slower vasodilation reflecting elevated vascular resistance linked to hypertension. Early blood pressure management is therefore essential to reduce the risk of radiation-induced CVD.

**Supplementary Information:**

The online version contains supplementary material available at 10.1186/s40959-025-00395-6.

## Background

Cardiovascular diseases (CVD) remain the leading cause of death worldwide [1]. Growing evidence suggests that exposure to ionizing radiation increases CVD risk, as initially observed in Japanese atomic bomb survivors and later confirmed in other exposed populations, including Chernobyl recovery workers and nuclear industry employees [2–5] revealing a dose-dependent excess risk for stroke and heart disease as well as hypertension and myocardial infarction even at low and moderate radiation doses [2, 6, 7]. Among cancer survivors, radiation therapy - despite its clear survival benefits - is associated with a significant rise in long-term cardiovascular complications including coronary artery disease, myocardial infarction, pericardial fibrosis, valvular stenosis, and microvascular damage [8, 9].

Radiation-induced cardiovascular damage is thought to originate from injury to the vascular endothelium, which plays a central role in maintaining vascular homeostasis. Ionizing radiation induces oxidative stress and inflammation through increased release of reactive oxygen species (ROS), proteases, and proinflammatory mediators that disrupt endothelial barrier integrity [10]. The resulting increase in endothelial permeability promotes the adhesion and transmigration of circulating monocytes into the subendothelial space, facilitating the accumulation of low-density lipoprotein (LDL) cholesterol, foam cell formation, intimal thickening, and ultimately, atherosclerotic plaque development [9, 11]. Chronic inflammation and endothelial dysfunction are recognized as key contributors not only to atherogenesis but also to arterial stiffening [12].

Early impairments in vascular flexibility such as arterial stiffness can be detected before the clinical manifestation of overt vascular disease, its measurement has emerged as a valuable tool and an independent predictor of cardiovascular morbidity and mortality – comparable to conventional risk factors such as age, blood pressure and diabetes [13, 14]. Elevated arterial stiffness, typically assessed by pulse wave velocity (PWV), has been documented in survivors of childhood cancers [15], breast cancer [16, 17], Hodgkin´s lymphoma [18] and head and neck cancer [19] suggesting that radiation exposure may promote arterial stiffening, reinforcing the relevance of early vascular assessment in irradiated populations.

To further elucidate vascular manifestations as early mechanisms of radiation-induced CVD, we investigated arterial flexibility in a preclinical mouse model. Specifically, we analyzed the Arteria saphena, modelling large peripheral vessels, in both wildtype C57BL/6 mice and atherosclerosis-prone apolipoprotein E-deficient (ApoE^−/−^) mice. ApoE is a liver-derived glycoprotein binding to low- and very low density lipoprotein (VLDL) receptors supporting the clearance of plasma chylomicrons and VLDL remnants [20]. While wildtype mice rarely develop spontaneous cardiovascular lesions, ApoE^−/−^ mice exhibit hypercholesterolemia and progressive atherosclerosis closely resembling human pathology [20–22], making them well suited for the study of human-like cardiovascular pathology [21, 23]. Notably, ApoE^−/−^ mice have been hypothesized to exhibit heightened vascular sensitivity to radiation-induced injury. Previous studies have demonstrated that irradiated ApoE^−/−^ arteries display increased thrombomodulin and tissue factor expression, along with macrophage-rich, inflammatory lesions absent in irradiated C57BL/6 mice [24, 25].

We employed Optical Coherence Tomography (OCT), a high-resolution, non-invasive imaging technique, to longitudinally monitor radiation-induced alterations in vascular function. Although traditionally applied in ophthalmology, OCT has demonstrated utility in detecting early microvascular changes in radiation retinopathy and radiation-induced coronary artery disease [26, 27]. The differentiation of atherosclerotic plaques and the measurement of lumen and vessel areas ex vivo using OCT has demonstrated very high accuracy compared to standard methods such as histopathology and intravascular ultrasound [28]. Here, we expand its application to characterize radiation-induced changes in vascular flexibility in vivo assessed by measuring arterial diameters and the speed of diameter changes during vasoconstriction and vasodilation aiming to provide potential biomarkers for arterial stiffness as an early event in the development of CVD to improve cardiovascular risk stratification and enable early, personalized preventive treatment strategies following radiation exposure.

## Methods

### Animals

The present study involved C57BL/6 wildtype female mice aged 7–8 weeks (*n* = 60) (Charles River, Germany) and sex- and age-matched ApoE knockout mice (*n* = 60) (B6.129P2Apoe^tm1Unc^/Crl, Charles River, Germany). Mice were group-housed in temperature- (22–24 °C) and humidity (50–60%)-controlled animal facilities with a 12 h light/dark cycle between 6:00 am − 6:00 pm. Pelleted food (ssniff^®^ R/M-H, V1534, ssniff Spezialdiäten GmbH, Soest, Germany) and water were available ad libitum.

### Irradiation

**Fig. 1:**
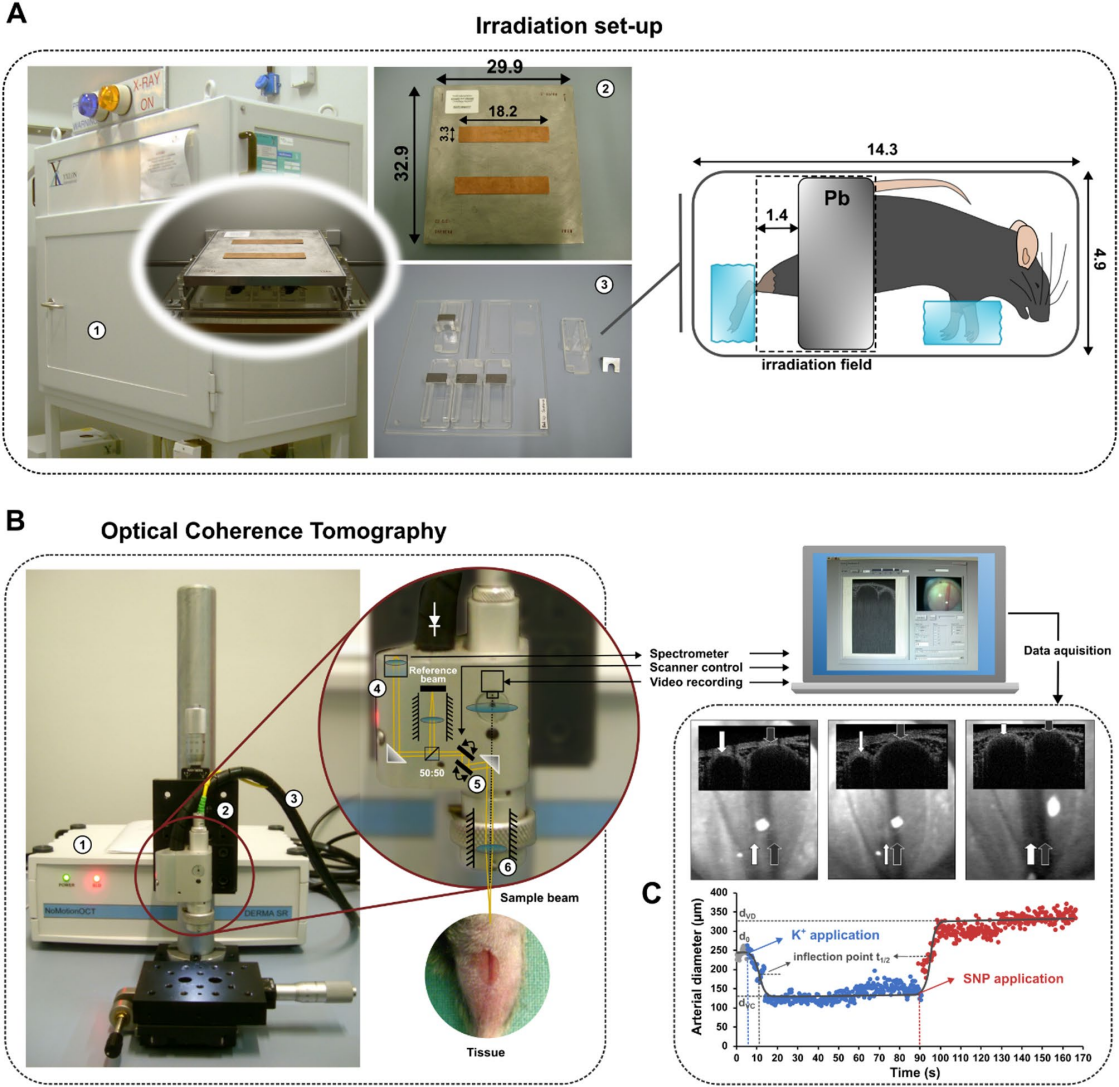
Experimental set-up for irradiation and OCT-imaging of the murine Arteria saphena. **A** For irradiation in the X-ray device (1), anesthetized animals were positioned on their left side and secured on a Plexiglas holder. The bent right leg and lower abdomen were shielded with lead to protect them from radiation, ensuring that only the inner side of the left lower leg remained within the irradiation field. The exposure area beneath the irradiation window was defined by a collimator plate made of a bismuth-lead-tin alloy (MCP-96) with copper cutouts (2). Up to five animals were irradiated simultaneously on an underlying Plexiglas plate (3). The mesures are given in centimeters. **B** The OCT system for vascular imaging of the A. saphena operates using near-infrared light emitted by a diode (1), which is transmitted to the scanner head (2) via fiber optic cables (3). Within the scanner head the incoming light is collimated to a beam of 2.4 mm in diameter through a collimator (4) (focal length = 12 mm) and subsequently divided into a reference and probe beam of equal diameter with a beam splitter. To scan the arterial surface, the probe beam is diffracted via two galvanometric scanners (5) (Cambridge Technologies, Planegg) and focused through an achromatic lense (6) (focal length = 25.4 mm, diameter = 15 mm). The light reflected by the arterial surface and the reference beam that has been reflected by a mirror are then recombined by the beam splitter. Fiber optic cables lead the resulting interference signal through a collimator (focal length = 40 mm) and to a spectrometer to be spectrally analyzed with a diffraction grating (1200 lines/mm). The interference spectrum is then focused through an achromatic lense (focal length = 75 mm) and detected with a silicon detector (LIS-1024, pixel size: 7.8 μm × 125 μm × 1024 px, Photon Vision Systems Inc., Homer, USA). A Fast Fourier Transform of the interference signal provides depth-resolved information about the arterial tissue. **C** Representative recording of the A. saphena (white arrows) and Vena saphena medialis (grey arrows) of a C57BL/6 mouse aged 8 weeks, one day after irradiation with 2 Gy. The upper picture row in the foreground represents 2-D cross sectional OCT-images. The picture row below in the background are video-recordings to orientate on the tissue. Left: Vessel diameter at rest after application of physiological buffer solution. Middle: Arterial vasoconstriction (VC) after application of buffer solution with high potassium concentration (K+). Right: Arterial vasodilation (VD) induced by sodium nitroprusside (SNP). The diameter of the saphenous vein was unaffected. Below: Time course of inner diameter changes of A. saphena with fitted sigmoid function (black line). d0: initial diameter, dVC: minimal diameter during VC. dVD: maximal diameter during VD. t_1/2_: time of half VC or VD

Animals (*n* = 12 per strain, batch and dose group, Additional file 1) were anaesthetized intraperitoneally with pentobarbitone sodium (60 mg/kg, Narcoren^®^, Merial GmbH, Hallbergmoos, Germany) and fixed in a custom-made Plexiglas^®^ device to expose the inner left leg to the radiation field (Fig. 1,A, left). The A. saphena was locally irradiated with single doses of 2, 5, 8, 10,16 Gy, respectively. The non-irradiated vessel of the right leg served as an individual control.

Irradiation was performed with vertical beam projection using an Yxlon MG 325 - Y.TU/320-D03 X-ray device (Yxlon International X-Ray GmbH, Hamburg, Germany) operating at a potential of 200 kV and at a current intensity of 20 mA. The dose rate at the focus–object distance of 48.5 cm was 0.8655 Gy/min.

During irradiation the animals were shielded using a collimator plate (32.9 cm × 29.9 cm x 1.1 cm, alloy MCP-96, HEK Medizintechnik, Lübeck, Germany) with rectangular windows (18.2 cm × 3.3 cm) consisting of 0.6 mm copper to define the radiation field. The left leg was further protected with a lead shielding restricting irradiation to the shank at a length of 1.4 cm (Fig. 1A).

### Optical coherence tomography

For each strain, OCT was conducted at 1 day and at 3, 6, 9, 12, and 18 months post-irradiation in five batches of up to 12 animals per radiation dose and time interval (Additional file 1).

To estimate vasodynamics of the A. saphena we used a Fourier Domain OCT system (NoMotion OCT – Derma SR) with customized LabView™ and MATLAB scripts [29] with a wavelength of 830 nm, a half-power width of 50 nm and a refractivity of 1 mW (Fig. 1B). A broadbent superluminescence diode (SLD-37-MP, Superlum Diodes Ltd., Moscow, Russia) served as light source. Image acquisition of the inner arterial diameter of the A. saphena was performed at a frame rate of 4 B-scans per second. Each B-scan is composed of 300 A-Scans in similar lateral position.

Prior to OCT animals were anaesthetized through intraperitoneal injection of pentobarbitone sodium (60 mg/kg, Narcoren^®^, Merial GmbH, Hallbergmoos, Germany) and fixed on the object table in the body position similar as during irradiation. Following depilation of the inside of a shank the A. saphena was exposed by removing a skin areal of approx. 5 × 5 mm^2^ close to the knee joint. To prevent excessive scarring of skin tissue, each batch of animals was examined at only two consecutive time points (Additional file 1).

The vessel was positioned within the light beam below the scanner head. A camera integrated into the scanner head enabled precise orientation above the artery. To assess the arterial diameter at baseline the exposed A. saphena was moistened with a physiological buffer solution (NaCl: 119 mmol/l, Merck, Darmstadt, Germany; KCl: 4.7 mmol/l, Merck; MgSO_4_: 1.17 mmol/l, Sigma-Aldrich, Taufkirchen, Germany; NaHCO_3_: 25 mmol/l, Merck; KH_2_PO_4_: 1.18 mmol/l, Merck; Glucose: 5.5 mmol/l, Merck; EDTA: 0.027 mmol/l, Prolabo, VWR International, Darmstadt) right before starting OCT. Acquisition of the baseline diameter stopped automatically after 30 initial B-scans (equivalent to a recording time of 7.5 s).

For subsequent induction of vasoconstriction, the baseline buffer solution was removed from the vessel and exchanged with a buffer of the same composition but with increased amount of KCl (80 mmol/l) before continuing OCT. Recording of the decreasing arterial diameter stopped again automatically after 300 B-scans (equivalent to an acquisition time of 75 s).

Finally, the buffer solution was removed and vasodilation was induced by application of sodium nitroprusside (1 mmol/l, Lachema, Chemapol AG, Prague, Czech Republic) before re-starting the OCT to assess the increasing arterial diameter within another 300 B-scans.

### Analysis of vasodynamics

An OCT image of the A. saphena revealed a half-round structure of the light grey vessel wall and a dark colored area corresponding to blood (Fig. 1C). Using the graphical programming system LabView™ (Version 8.2., 2006, National Instruments, Austin, USA) the boundary between blood and vessel wall was marked as a circle and set as region of interest (ROI) within the first B-scan. Based on this ROI the inner arterial diameters were automatically assessed during all subsequent B-scans. Subsequent analysis of vasodynamics using the recorded diameter values (d(t)) with corresponding time points (t) was performed using GraphPad Prism (Version 5.04, GraphPad Software Inc., La Jolla, San Diego, USA). Applying a sigmoidal curve function

$$\:d\left(t\right)={d}_{VC}+\frac{({d}_{VD}-{d}_{VC})}{1+{10}^{\left({t}_{1/2}-t\right)\bullet\:Hillslope}}$$.

minimal (d_VC_) and maximal (d_VD_) arterial diameters during vasoconstriction (VC) and vasodilation (VD) as well as the time to half of arterial diameter change (t_1/2_) were calculated. The Hillslope indicates the steepness of the curve.

Statistical analyses were carried out using R (Version 4.2.3, R Foundation for Statistical Computing [30] applying a multifactorial analysis of variance (ANOVA) with factors strain, dose and time after irradiation. Since significant Levene’s tests indicated inhomogenious variances, we chose Games-Howell post-hoc tests (α = 0.05) for multiple comparisons of dose groups, post-irradiation time intervals and interactions between the factors.

## Results

**Fig. 2:**
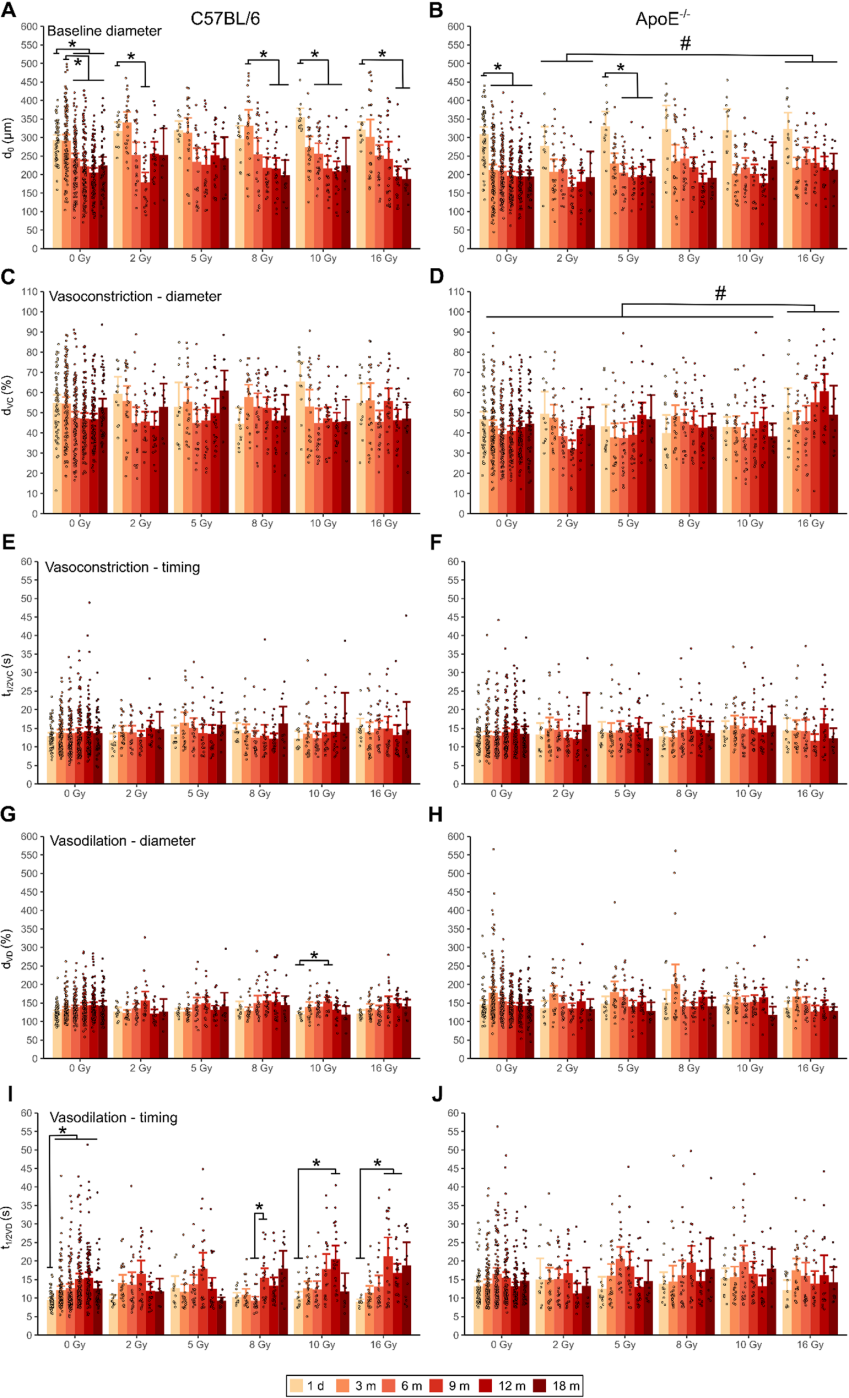
Vascular parameters of the murine Arteria saphena assessed by Optical Coherence Tomography.** A**,** B** Initial baseline arterial diameters (d_0_), **C**,** D**) minimal arterial diameters during vasoconstriction (VC) (d_VC_), **E**,** F**) time of half d_VC_, **G**,** H**) maximal arterial diameters during vasodilation (VD) (d_VD_) and **I**,** J**) time of half d_VD_ in C57BL/6 wildtype (left panels) and ApoE^−/−^ mice (right panels), recorded 1 day and 3, 6, 9, 12, 18 months following irradiation of the inner left leg with 2, 5, 8, 10, 16 Gy. The non-irradiated inner right legs (0 Gy) served as individual controls. Data are displayed as mean barplots ± 95% confidence intervals with individual datapoints. * = significant effects of age, # = significant effects of dose within each strain. Further analyses incl. precise *p*-values are given in Additional file 3

The initial baseline diameters of the un-irradiated A. saphena of C57BL/6- and ApoE^−/−^ mice were 293 ± 52 μm and 309 ± 73 μm (mean ± SD), respectively (Fig. 2A, B, Additional file 2). At 3 months post-irradiation, a systematic reduction in baseline diameters was observed in ApoE^−/−^ mice only, and from 6 months in both strains as confirmed by ANOVA (main effect of time: *F*_5,2141_ = 68.891, *p* < 0.001, Additional file 3: Anova_baseline, Baseline_posthoc_time, Baseline_posthoc_strain_time) revealing a significant effect of increasing age. Likewise significant effects of strain (*F*_1,2141_ = 61.172, *p* < 0.001) and interaction with age (*F*_5,2141_ = 12.865, *p* < 0.001, Additional file 3: Anova_baseline, Baseline_posthoc_strain_time) further indicate that this effect was more pronounced in ApoE^−/−^ mice. Significant interaction effects with radiation dose point to an increased radiation susceptibility of ApoE ^−/−^mice (*F*_5,2141_ = 2.799, *p* < 0.05, Additional file 3: Anova_baseline, Baseline_posthoc_strain_dose) as well as accumulating effects of radiation and age in older animals of both strains (dose: time interaction: *F*_25,2141_ = 1.878, *p* < 0.01, Additional file 3: Anova_baseline, Baseline_posthoc_dose_time).

Induction of vasoconstriction resulted in arterial diameters to on average about half of baseline values in C57BL/6- and even less in ApoE^−/−^ mice (Fig. 2C, D, Additional file 2) (main effect of strain: *F*_1,2141_ = 67.936, *p* < 0.001, Additional file 3: Anova_min, Min_posthoc_strain_dose) indicating an enhanced vasoconstrictive response in the latter, which however was affected by radiation with 16 Gy (main effect of dose: *F*_5,2141_ = 2.918, *p* < 0.05, strain: dose interaction: *F*_5,2141_ = 2.641, *p* < 0.05, Additional file 3: Anova_min, Min_posthoc_dose, Min_posthoc_strain_dose). Increased contractility was also found to be age-dependent (*F*_5,2141_ = 2.641, *p* < 0.001, Additional file 3: Anova_min, Min_posthoc_time) as revealed by the most pronounced reduction in arterial diameters between 6 and 12 months post-irradiation especially in C57BL/6 mice (strain: time interaction: *F*_5,2141_ = 5.791, *p* < 0.001, Additional file 3: Anova_min, Min_posthoc_strain_time). Time to half-minimal arterial diameter during vasoconstriction (Fig. 2E, F, Additional file 2) in un-irradiated vessels was initially 12.9 ± 3.4 s (mean ± SD) in C57BL/6- and 13.0 ± 4.0 s in ApoE^−/−^ mice with no significant differences between strains, radiation doses or time intervals after irradiation (Additional file 3: Anova_Tmin).

Maximal arterial diameters following induction of vasodilation (Fig. 2G, H, Additional file 2) were on average in the range of 1.2–2-fold the baseline with higher values in ApoE^−/−^ mice (main effect of strain: *F*_1,2131_ = 29.987, *p* < 0.001, Additional file 3: Anova_max) that declined with age after an initial increase at 3 months after irradiation (main effect of time: *F*_5,2131_ = 6.577, *p* < 0.001, strain: time interaction: *F*_5,2131_ = 16.037, *p* < 0.001, Additional file 3: Anova_max, Max_posthoc_time, Max_posthoc_strain_time). In contrast, C57BL/6 mice displayed an enhanced vasodilation up to 12 months after irradiation. An effect of radiation dose was not observed. Time to half-maximal arterial diameter during vasodilation (Fig. 2I, J, Additional file 2) in un-irradiated vessels was initially 9.4 ± 2.2 s in C57BL/6 and 13.1 ± 5.2 s in ApoE^−/−^ mice that displayed generally longer enduring vasodilation (main effect of strain: *F*_1,2115_ = 24.851, *p* < 0.001, Additional file 3: Anova_Tmax). Vasodilation slowed down in aging animals (main effect of time: *F*_5,2115_ = 13.819, *p* < 0.001, Additional file 3: Anova_Tmax, Tmax_posthoc_time) especially in C57BL/6 mice (strain: time interaction: *F*_5,2115_ = 9.673, *p* < 0.001, Additional file 3: Tmax_posthoc_strain_time) and most pronounced at 9 and 12 months following irradiation with 10 Gy and 16 Gy (dose: time interaction: *F*_25,2115_ = 1.756, *p* < 0.05, strain: dose: time interaction: *F*_25,2115_ = 1.628, *p* < 0.05, Additional file 3: Anova_Tmax, Tmax_posthoc_time, Tmax_posthoc_strain_dose_time).

## Discussion

Aging has been described as the primary driver of arterial stiffness [12]. However, the increased incidence of arterial stiffness and subsequent cardiovascular complications observed in cancer patients that received radiotherapy [31–34], combined with the fact that arterial stiffness is still underutilized as a prognostic indicator in clinical practice [35], highlights the need to assess the impact of radiation exposure on vascular compliance. This is particularly relevant as increased arterial stiffness has also been linked to a higher risk of cancer recurrence [36].

It has been hypothesized that aging and radiation share overlapping mechanisms that exacerbate vascular damage. Both contribute to the accumulation of ROS, leading to excessive oxidative stress that damages DNA, proteins, and membrane lipids while simultaneously disturbing the nuclear organization and damage repair. Furthermore, aging-related telomere attrition has been associated with increased radiosensitivity, as cells with shortened telomeres exhibit impaired repair of radiation-induced DNA breaks and enhanced chromosome instability [37, 38]. Radiation has also been shown to promote cellular senescence of vascular endothelial cells, a process believed to contribute to the pathogenesis of cardiovascular diseases following radiation exposure [39].

Given these overlapping and potentially synergistic mechanisms, we investigated vascular biomarkers aiming to differentiate age- and radiation-related contributions to increased arterial stiffness as an early event of CVD. We used a Fourier Domain-OCT system for in vivo longitudinal assessment of vascular function of the murine saphenous artery up to 18 months following local irradiation with single doses between 2 and 16 Gy. We have chosen the murine A. saphena as a model of large arterial vessels as it is easily accessable due to its optimal anatomical position at the inner hind legs beneath the skin and the musculus vastus medialis. It offers inner arterial diameters of suitable, well measurable sizes and a sensitive response to vasoactive substances. The OCT system allowed contact-free imaging of the A. saphena requiring only superficial access to the vessel. Appropriate resolution and scan rate of the OCT system allowed to measure inner arterial diameter changes precisely and enabled following the time course of vasoconstriction and vasodilation. Compared to PWV — the current gold standard for assessing arterial stiffness based on the speed of pressure wave propagation along a vessel [40, 41] — OCT offers superior spatial and temporal resolution and enables direct visualization of local vascular behavior, independent of blood pressure and vascular tone [29]. However, PWV has greater tissue penetration, and while its assessment of average stiffness across arterial segments may mask local dysfunction, it provides a broader, regional [42] or even systemic [43] evaluation of vascular impairment. Since both techniques offer distinct yet complementary insights, their combined application could enhance the comprehensive assessment of vascular function.

While age-related vascular dysfunction was evident in both C57BL/6 wildtype and ApoE^−/−^ mice, the latter displayed an earlier decline, with reductions in baseline arterial diameters apparent from 3 months post-irradiation. ApoE^−/−^ mice, a recognized model for human CVD, further exhibited heightened radiosensitivity reflected in greater reductions in baseline diameters and minimal diameters after vasoconstriction, alongside prolonged vasodilation times. In line, previous studies using a similar OCT setup demonstrated that both vasoconstriction and vasodilation durations increase with age in mice [29, 44]. This effect was further amplified by a high-fat diet, another risk factor for endothelial dysfunction [44]. Moreover, in hypercholesterolemic LDL receptor knockout mice, both minimal and maximal diameters of the A. saphena were reduced [45], indicating enhanced vasoconstriction and impaired vasodilation - paralleling the vascular alterations we observed in our ApoE^−/−^ mice.

Oxidative stress and vascular damage triggered by risk factors such as radiation exposure disrupt the balance between vasoconstrictors - including endothelin-1 and thromboxane A2 - and vasodilators such as nitric oxide (NO) and prostacyclin, ultimately favoring vasoconstriction [46–48]. Oxidative stress further promotes vasoconstriction by stimulating calcium release from intracellular stores in vascular smooth muscle cells (VSMCs) and by enhancing VSMC proliferation, either through secretion of the chaperone protein cyclophilin A or via interaction with oxidative stress byproducts such as hydroperoxyoctadecadienoic acids and 4-hydroxy-2-nonenal [49]. Pathological tissue remodeling due to fibrosis resulting from radiation-induced oxidative stress, inflammation, and vascular injury can further contribute to reduced vascular elasticity, narrowed arterial lumens, and increased vasoconstriction [50, 51]. Indeed, fibrotic markers have been associated with the progression of large-vessel atherosclerosis and the development of peripheral artery disease [52]. However, in the irradiated murine A. saphena, although several dose- and age-dependent proinflammatory responses were detected – such as increased expression of adhesion molecules CD31, E-selectin, ICAM-1, and VCAM-1, even at doses as low as 2 Gy – these effects appeared transient and did not lead to sustained histopathological changes indicative of fibrosis [53].

Impaired endothelium-dependent vasodilation is a key consequence of endothelial dysfunction, primarily due to reduced NO bioavailability. NO, the principal endothelium-derived relaxing factor, plays a central role in regulating vascular tone and reactivity [54]. Following radiation exposure, NO is rapidly inactivated by superoxide radicals, leading to the formation of vasotoxic peroxynitrite [49, 55]. Radiation-induced oxidative stress also causes uncoupling of endothelial nitric oxide synthase (eNOS) by depleting its redox-sensitive cofactor tetrahydrobiopterin, which shifts eNOS activity toward superoxide production instead of NO synthesis [49]. Clinically, diminished endothelium-dependent vasodilation has been observed in irradiated arteries years after radiotherapy, as demonstrated in breast cancer patients [56].

This altered vascular reactivity - marked by enhanced vasoconstriction and delayed vasodilation - may elevate peripheral vascular resistance, potentially contributing to sustained hypertension [57]. However, we acknowledge that systemic blood pressure was not measured in this study, and our findings are based on local changes in the A. saphena. While these alterations reflect impaired vascular function at the peripheral level, their translation into systemic hypertension remains to be confirmed. Enhanced peripheral vascular resistance, if unmanaged, may predispose to the known chronic conditions such as coronary artery disease, aneurysms, stroke, aortic dissection, congestive heart failure, and peripheral vascular disease [57]. Although timely identification and management of vascular abnormalities could delay or prevent severe clinical outcomes, current guidelines do not yet recommend routine cardiovascular screening after radiotherapy, and little is known about the best strategies for risk evaluation, including the ideal population and screening modality [58]. Vascular biomarkers, such as measures of vascular flexibility and arterial stiffness in particular, could improve identifying those at risk. Together with early intervention using antihypertensive agents - such as alpha- or beta-blockers, calcium channel blockers, diuretics, or angiotensin-converting enzyme inhibitors - this may ultimately reduce long-term cardiovascular complications in cancer survivors [35, 57].

## Conclusion

In summary, both mouse strains exhibited pronounced age-related vascular changes, with ApoE^−/−^ mice displaying an enhanced radiosensitivity. Importantly, in both strains, the combined effects of aging and radiation were most evident in reductions of arterial diameters at baseline and after vasoconstriction, as well as in the slowed speed of vasodilation. These parameters, which reflect heightened vasoconstriction and impaired vasodilation, are indicative of increased vascular resistance contributing to hypertension. Incorporating vascular flexibility assessments into post-treatment screening programs, alongside early management of elevated blood pressure, may help mitigate the risk of radiation-induced cardiovascular diseases.

## Supplementary Information

Additional file 1: Fig. S1 Experimental schedule for irradiation and vascular imaging using Optical Coherence Tomography (OCT)

Additional file 2: Descriptive statistics for baseline, minimal and maximal diameters of the A. saphena and timing of diameter changes in C57BL/6 and ApoE^-/-^ mice

Additional file 3: ANOVAs and post-hoc tests extracting significant differences of baseline, minimal and maximal diameters of the A. saphena and timing of diameter changes depending on mouse strain, radiation dose and time after irradiation

## Data Availability

The datasets generated during and/or analysed during the current study are available from the corresponding author on reasonable request.

## Abbreviations

ANOVA: analysis of variance
ApoE: apolipoprotein E
CVD: cardiovascular diseases
eNOS: nitric oxide synthase
LDL: low density lipoprotein
NO: nitric oxide
OCT: Optical Coherence Tomography
PWV: pulse wave velocity
ROI: region of interest
ROS: reactive oxygene species
VC: vasoconstriction
VD: vasodilation
VLDL: very low density lipoprotein
VSMC: vascular smooth muscle cell
