# Supplementary material for: Radiation- and age-related vascular dysfunction as an early indicator of cardiovascular risk: a long-term study in the ApoE−/− mouse model of atherosclerosis
Source: Cardiooncology. 2025 Oct 21;11:93. doi: 10.1186/s40959-025-00395-6 (PMC12538826; doi:10.1186/s40959-025-00395-6)
Supplement: Supplementary file 1 — Fig. S1 Experimental schedule for irradiation and vascular imaging using Optical Coherence Tomography (OCT) [file 40959_2025_395_MOESM1_ESM.pdf]

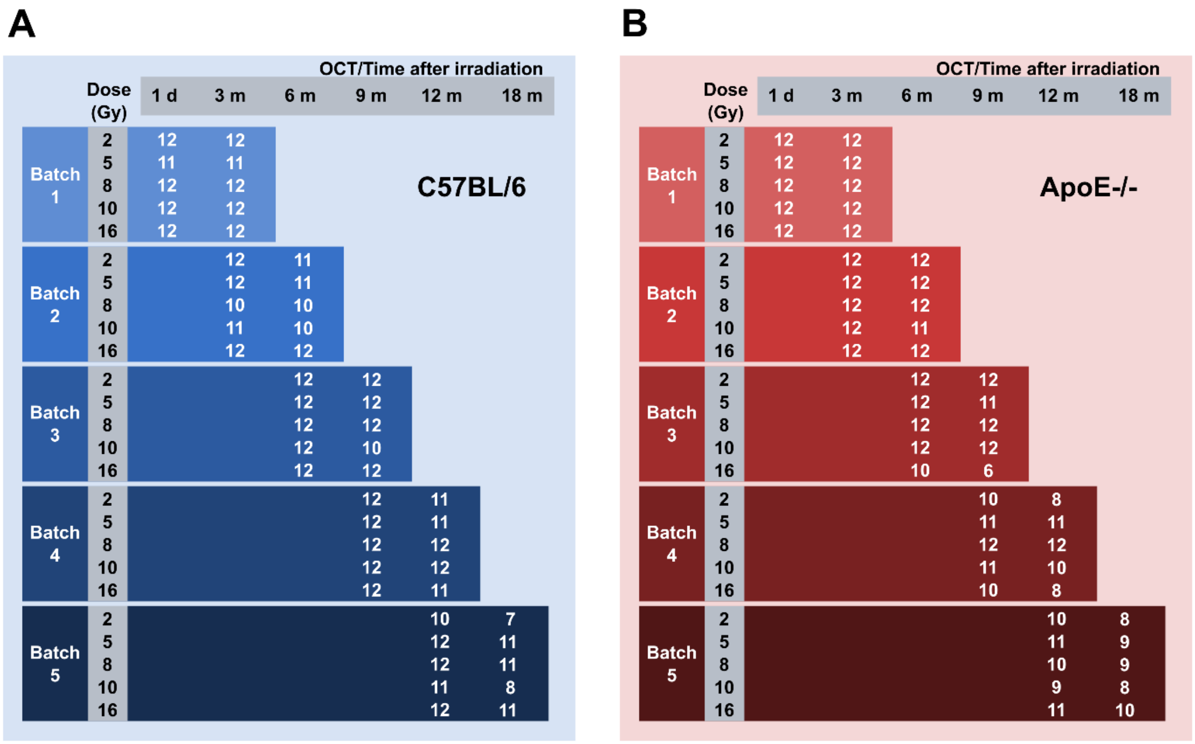

Fig. S1 Experimental schedule for irradiation and vascular imaging using Optical Coherence Tomography (OCT). Each experimental group for **A)** C57BL/6 and **B)** ApoE$^{-/-}$ mice comprised initially 12 animals per radiation dose and time point of OCT. The inner side of the left hind leg was subjected to irradiation with single doses of 2, 5, 8, 10, or 16 Gy. The inner side of the non-irradiated right hind leg served as individual control. OCT-Imaging of the A. saphena of both legs was conducted 1 day and 3, 6, 9, 12, and 18 months after irradiation, with two consecutive examination time points per batch. Animal losses occurred due to technical difficulties or mortality.
